# Supplementary material for: Five New Cucurbitane-Type Triterpenoid Glycosides from the Rhizomes of Hemsleya penxianensis with Cytotoxic Activities
Source: Molecules. 2019 Aug 13;24(16):2937. doi: 10.3390/molecules24162937 (PMC6720345; doi:10.3390/molecules24162937)
Supplement: Supplementary file 1 [file molecules-24-02937-s001.pdf]

Five new cucurbitane-type triterpenoid glycosides from the rhizomes of Hemsleya penxianensis with cytotoxic activities

De-Li Chen [1, 2†], Xu-Dong Xu [2†], Rong-Tao Li [1], Bo-Wen Wang[3], Meng Yu [1,2], Yang-Yang Liu [1,*], Guo-Xu Ma [1, 2,*]

[1] Hainan Branch of Institute of Medicinal Plant Development, Chinese Academy of Medicinal Sciences & Peking Union Medical College (Hainan Provincial Key Laboratory of Resources Conservation and Development of Southern Medicine), Haikou, China, 570311; chendeli9999@163.com;
[2] Institute of Medicinal Plant Development, Chinese Academy of Medical Sciences & Peking Union Medical College, No. 151, Malianwa North Road, Haidian District, Beijing, China, 100193; mgxfl8785@163.com;
[3] School of Chemical Engineering and Technology, Hainan University, Haikou, China, 570228 ; jasminewong@hainu.edu.cn
* Correspondence: eadchris@163.com; mgxfl8785@163.com; Tel.: +86-010-57833296
† Contributed equally to this work.

List of Figures S1-S29

Figure S1. ^{1}H-NMR (600 MHz, pyridine-d_5) spectrum of the new compound **1**

Figure S2. ^{13}C-APT (150 MHz, pyridine-d_5) spectrum of the new compound **1**

Figure S3. HSQC spectrum of the new compound **1**

Figure S4. HMBC spectrum of the new compound **1**

Figure S5. ^{1}H-^{1}H COSY spectrum of the new compound **1**

Figure S6. NOESY spectrum of the new compound **1**

Figure S7. ^{1}H-NMR (600 MHz, pyridine-d_5) spectrum of the new compound **2**

Figure S8. ^{13}C-APT (150 MHz, pyridine-d_5) spectrum of the new compound **2**

Figure S9. HSQC spectrum of the new compound **2**

Figure S10. HMBC spectrum of the new compound **2**

Figure S11. NOESY spectrum of the new compound **2**

Figure S12. ^{1}H-^{1}H COSY spectrum of the new compound **2**

Figure S13. ^{1}H-NMR (600 MHz, pyridine-d_5) spectrum of the new compound **3**

Figure S14. ^{13}C-APT (150 MHz, pyridine-d_5) spectrum of the new compound **3**

Figure S15. HMBC spectrum of the new compound **3**

Figure S16. NOESY spectrum of the new compound **3**

Figure S17. ^{1}H-^{1}H COSY spectrum of the new compound **3**

Figure S18. ^{1}H-NMR (600 MHz, pyridine-d_5) spectrum of the new compound **4**

Figure S19. ^{13}C-APT (150 MHz, pyridine-d_5) spectrum of the new compound **4**

Figure S20. HSQC spectrum of the new compound **4**

Figure S21. HMBC spectrum of the new compound **4**

Figure S22. NOESY spectrum of the new compound **4**

Figure S23. ^{1}H-^{1}H COSY spectrum of the new compound **4**

Figure S24. ^{1}H-NMR (600 MHz, pyridine-d_5) spectrum of the new compound **5**

Figure S25. ^{13}C-APT (150 MHz, pyridine-d_5) spectrum of the new compound **5**

Figure S26. HSQC spectrum of the new compound **5**

Figure S27. HMBC spectrum of the new compound **5**

Figure S28. NOESY spectrum of the new compound **5**

Figure S29. ^{1}H-^{1}H COSY spectrum of the new compound **5**

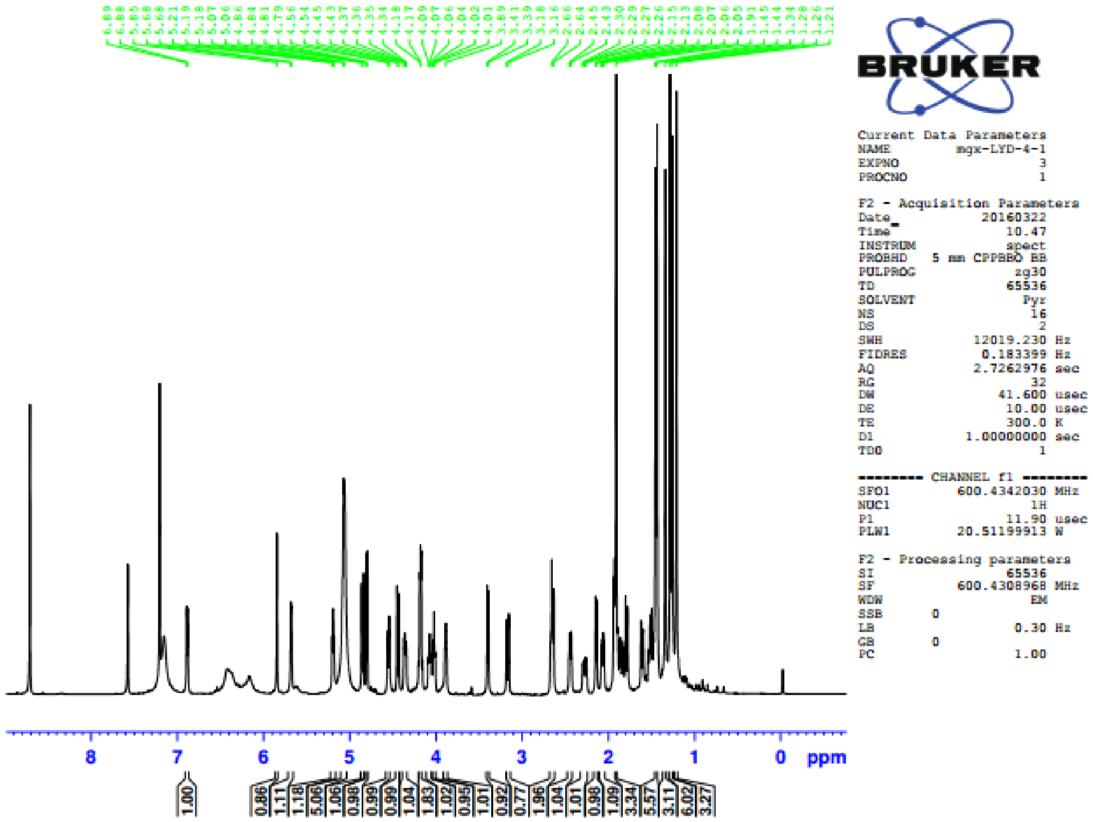

Figure S1. ^{1}H-NMR (600 MHz, pyridine-d_5) spectrum of the new compound **1**

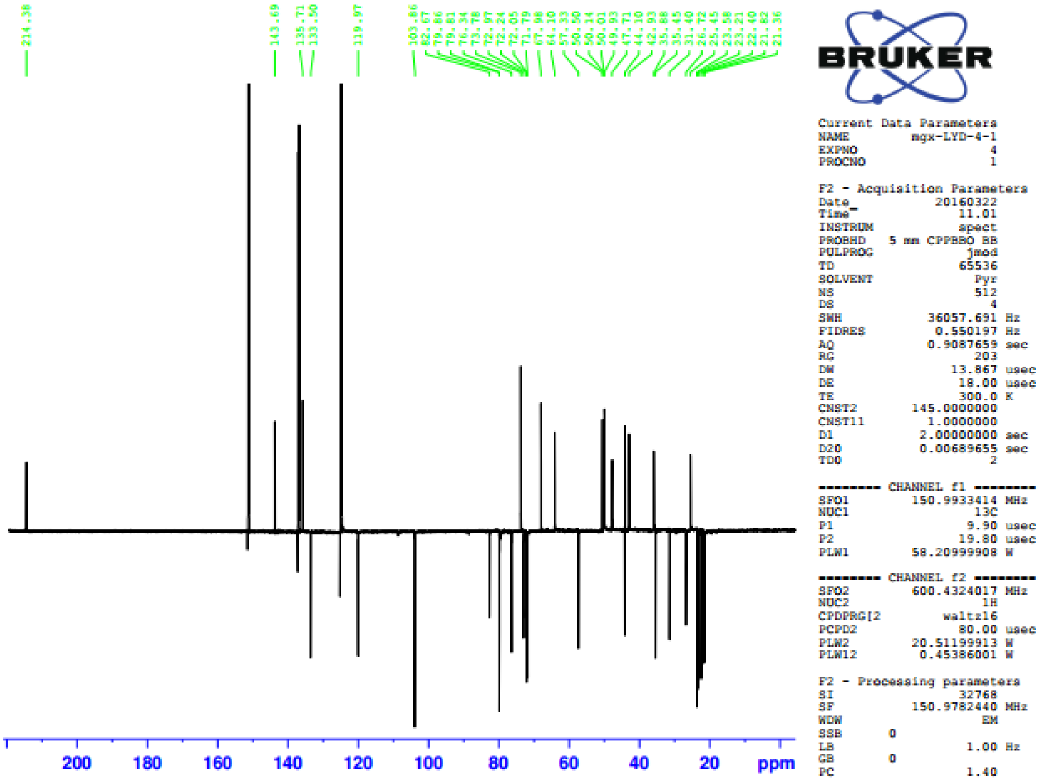

Figure S2. ^{13}C-APT (150 MHz, pyridine-d_5) spectrum of the new compound **1**

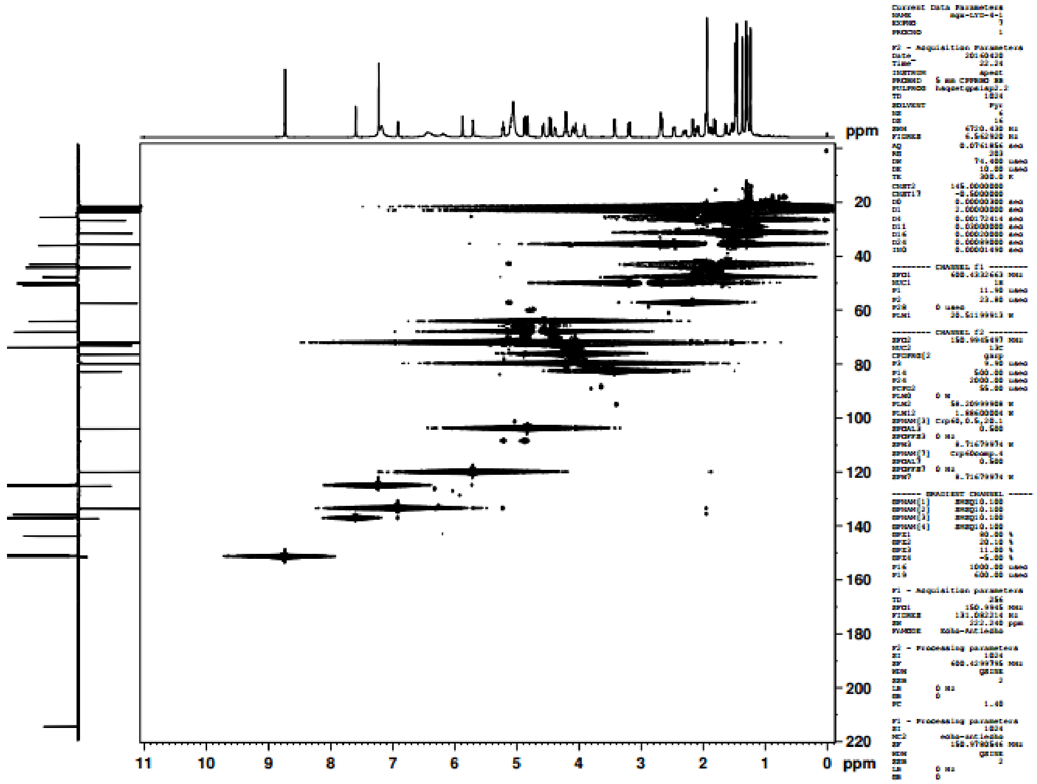

Figure S3. HSQC spectrum of the new compound **1**

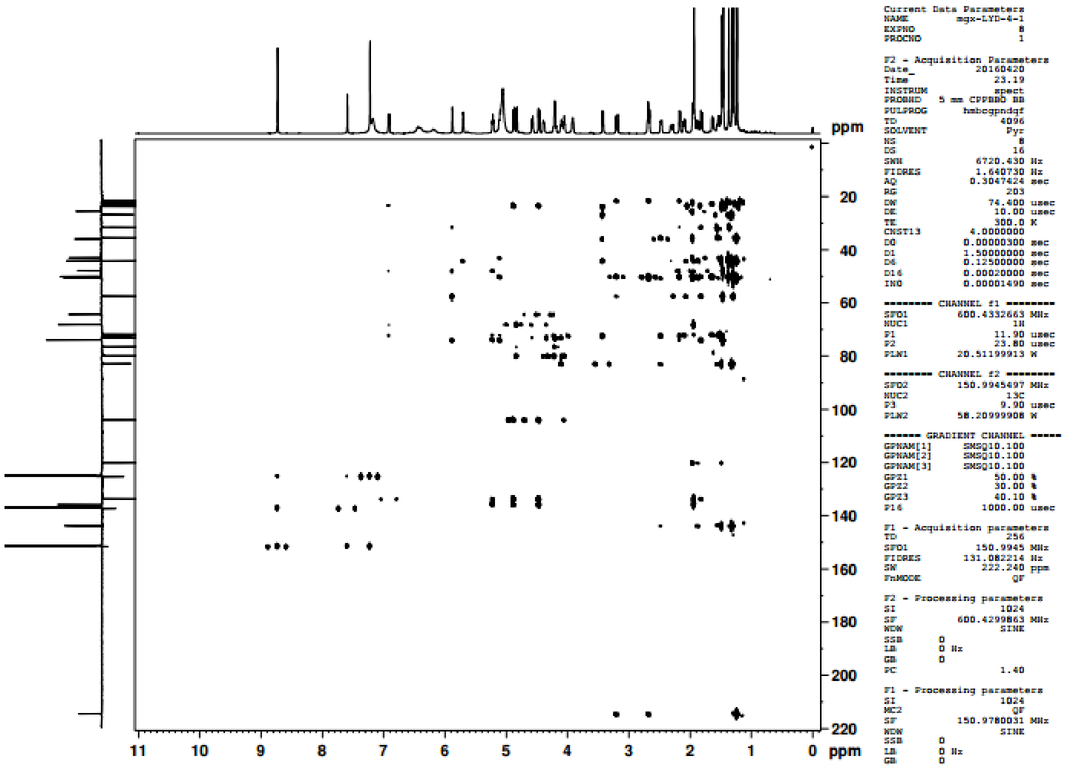

Figure S4. HMBC spectrum of the new compound **1**

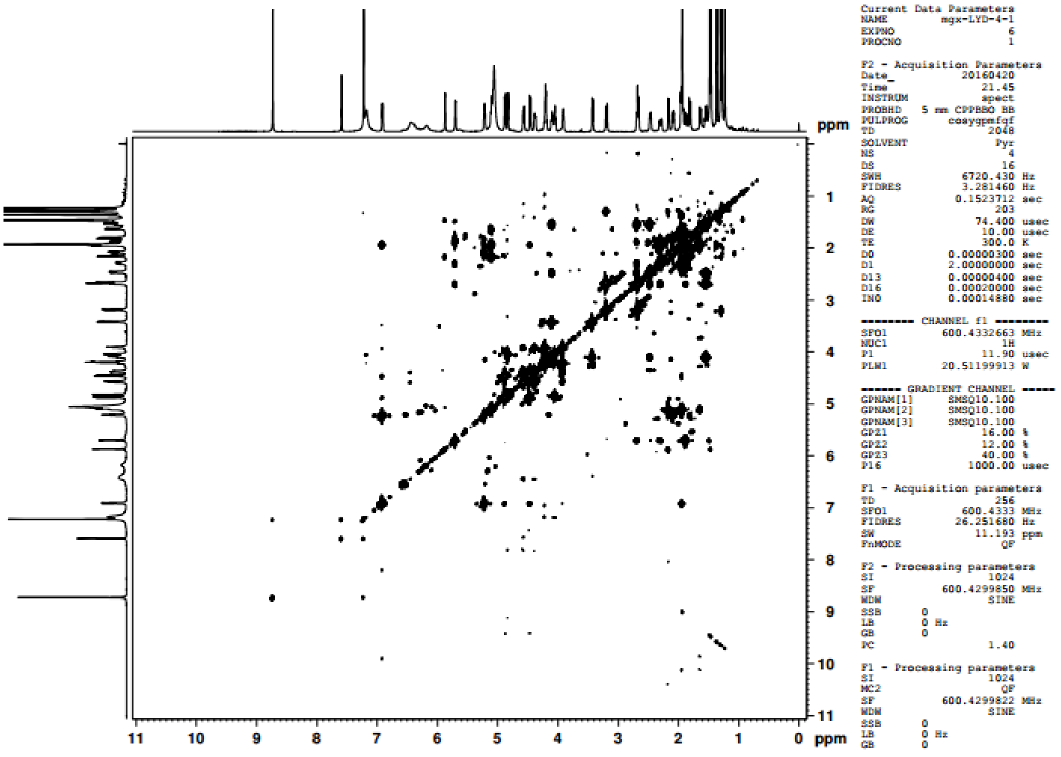

Figure S5. ¹H-¹H COSY spectrum of the new compound **1**

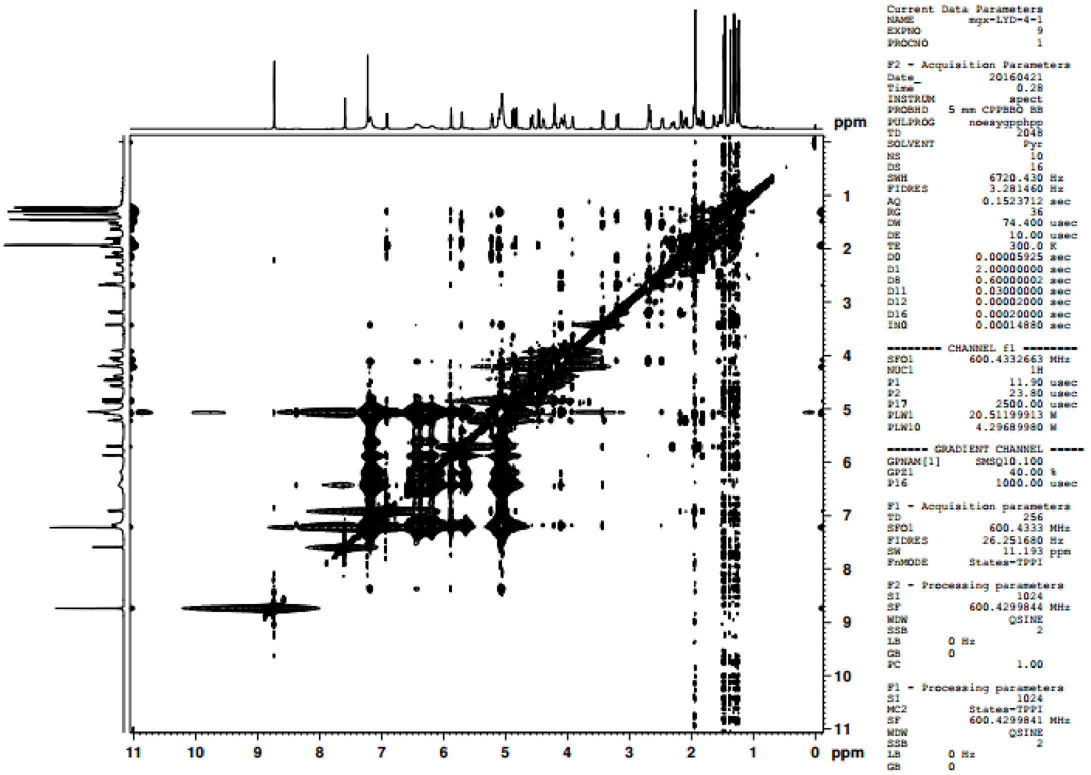

Figure S6. NOESY spectrum of the new compound **1**

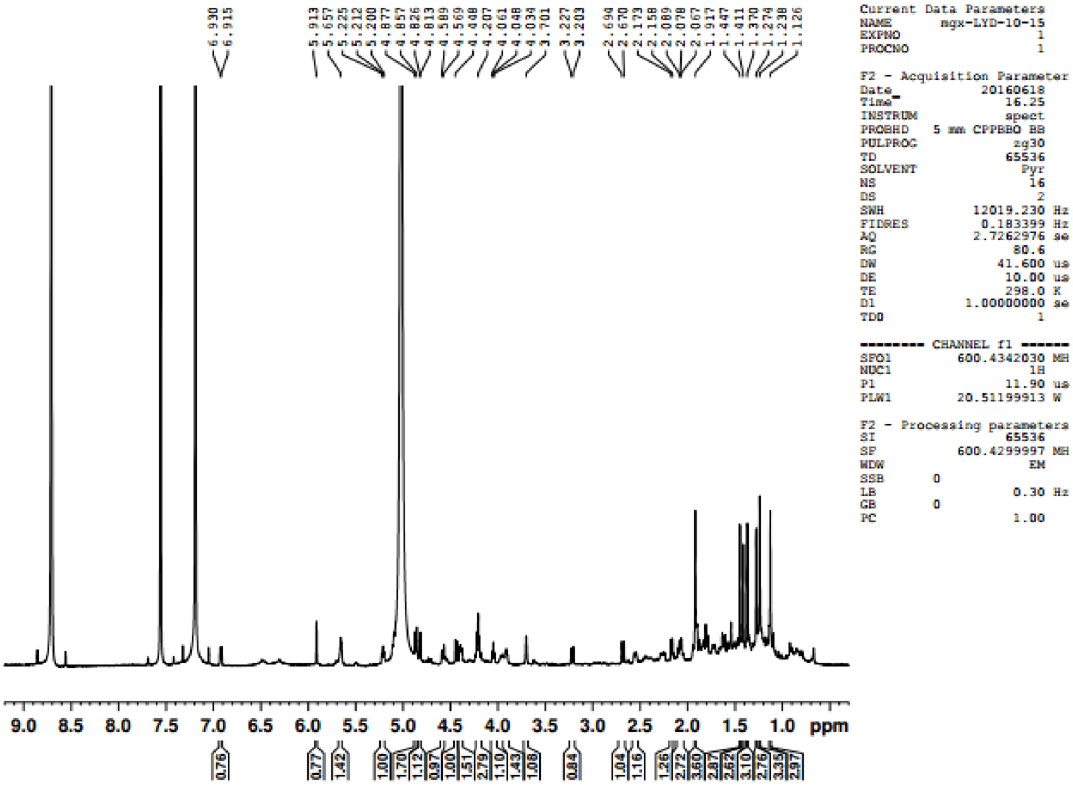

Figure S7. ^{1}H-NMR (600 MHz, pyridine-d_5) spectrum of the new compound **2**

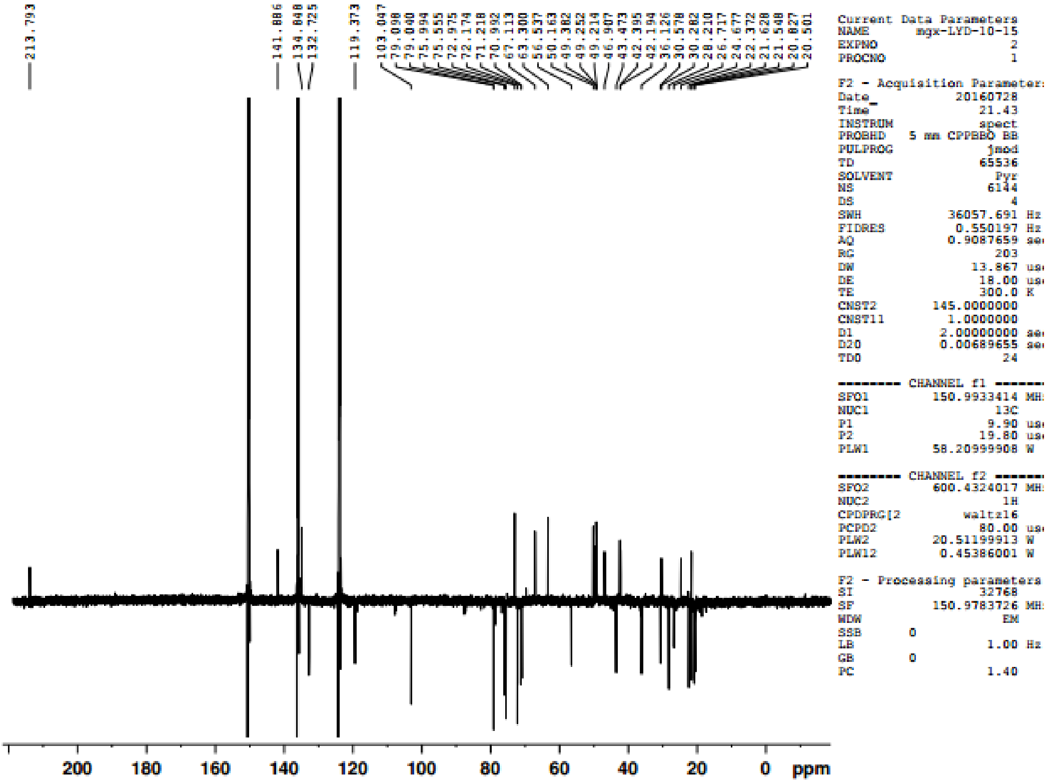

Figure S8. ^{13}C-APT (150 MHz, pyridine-d_5) spectrum of the new compound **2**

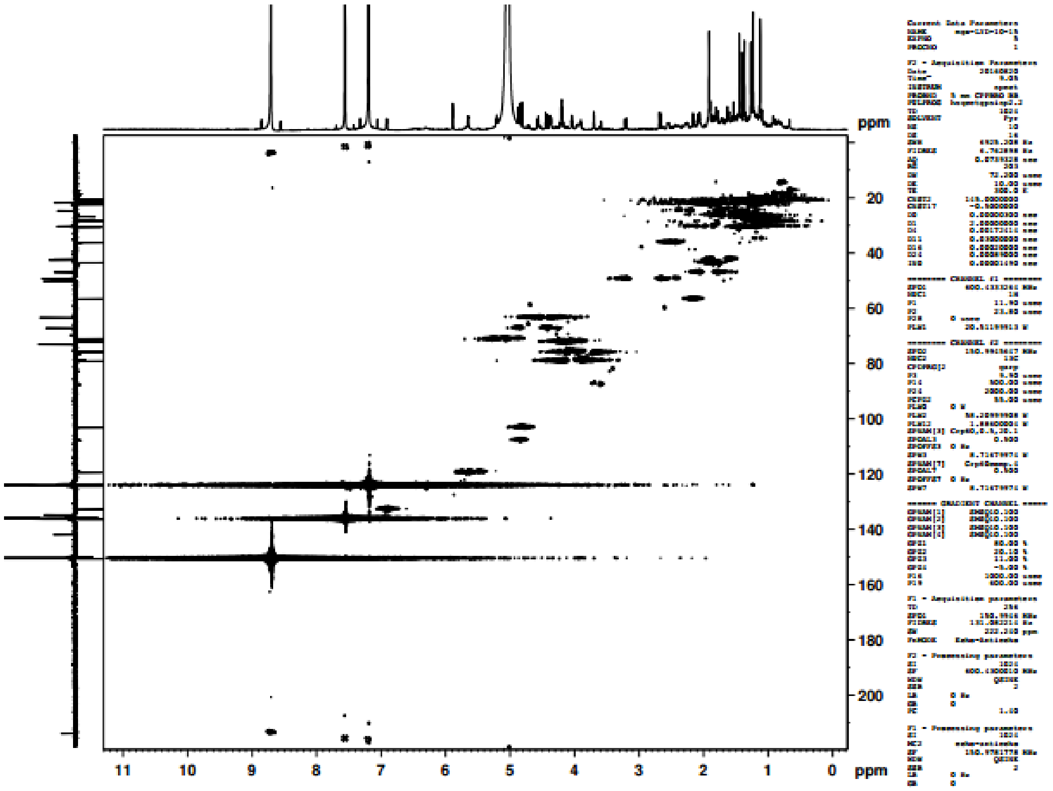

Figure S9. HSQC spectrum of the new compound **2**

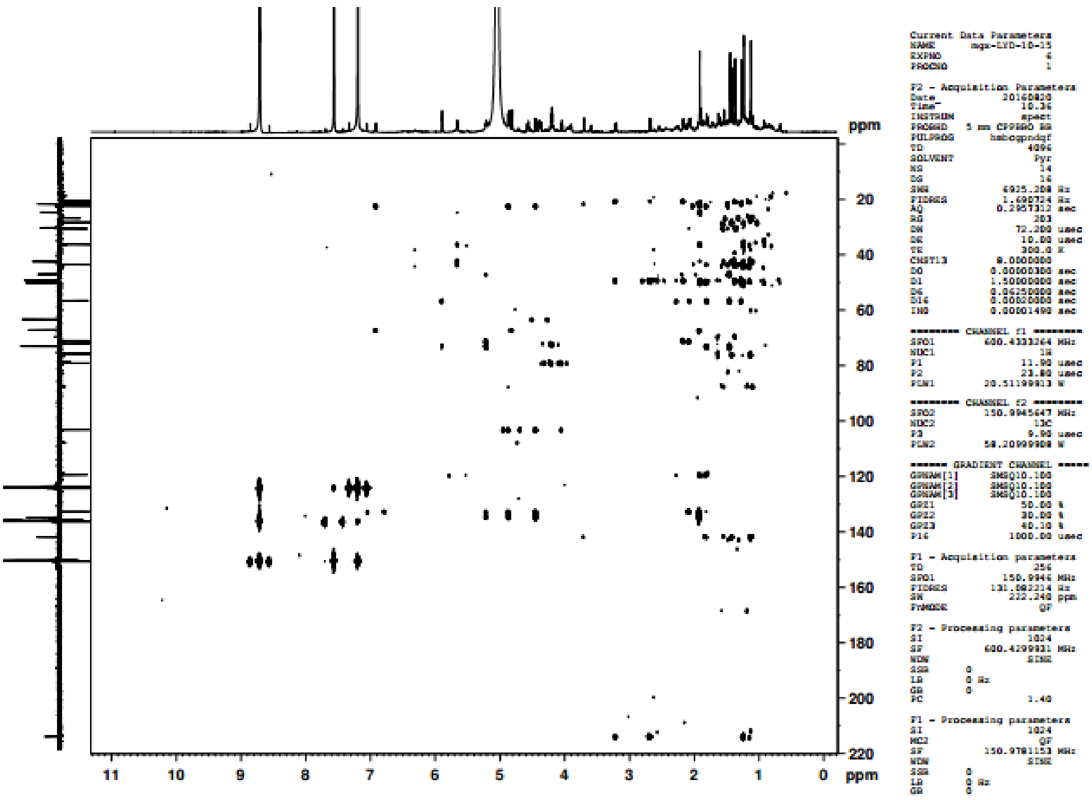

Figure S10. HMBC spectrum of the new compound **2**

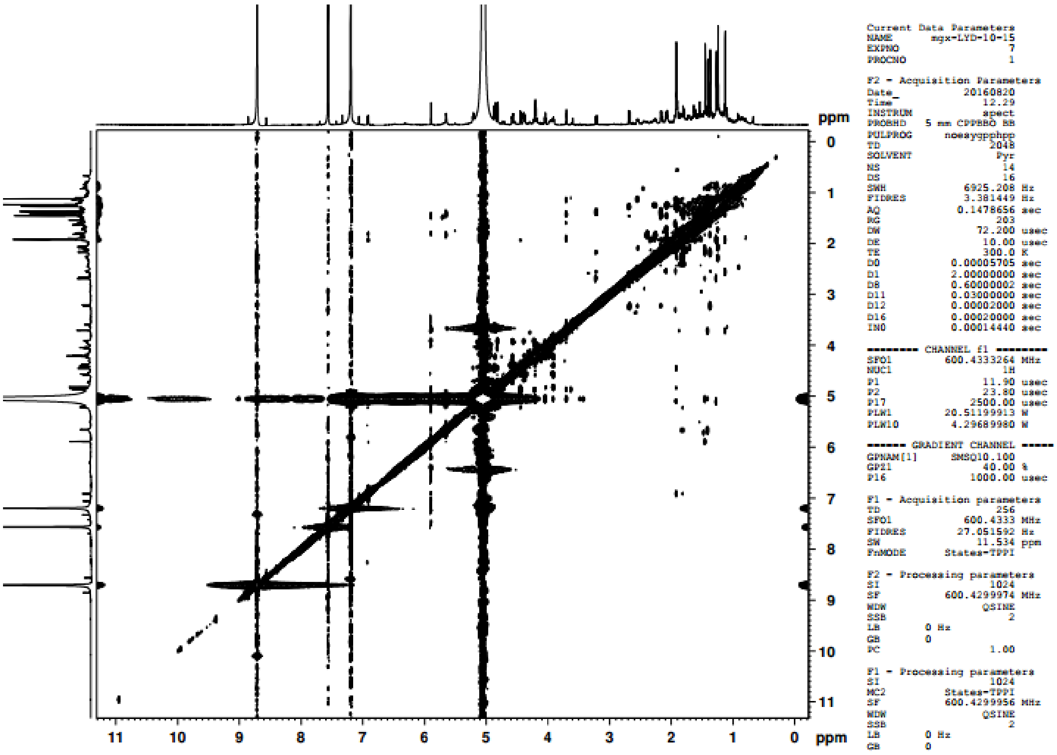

Figure S11. NOESY spectrum of the new compound **2**

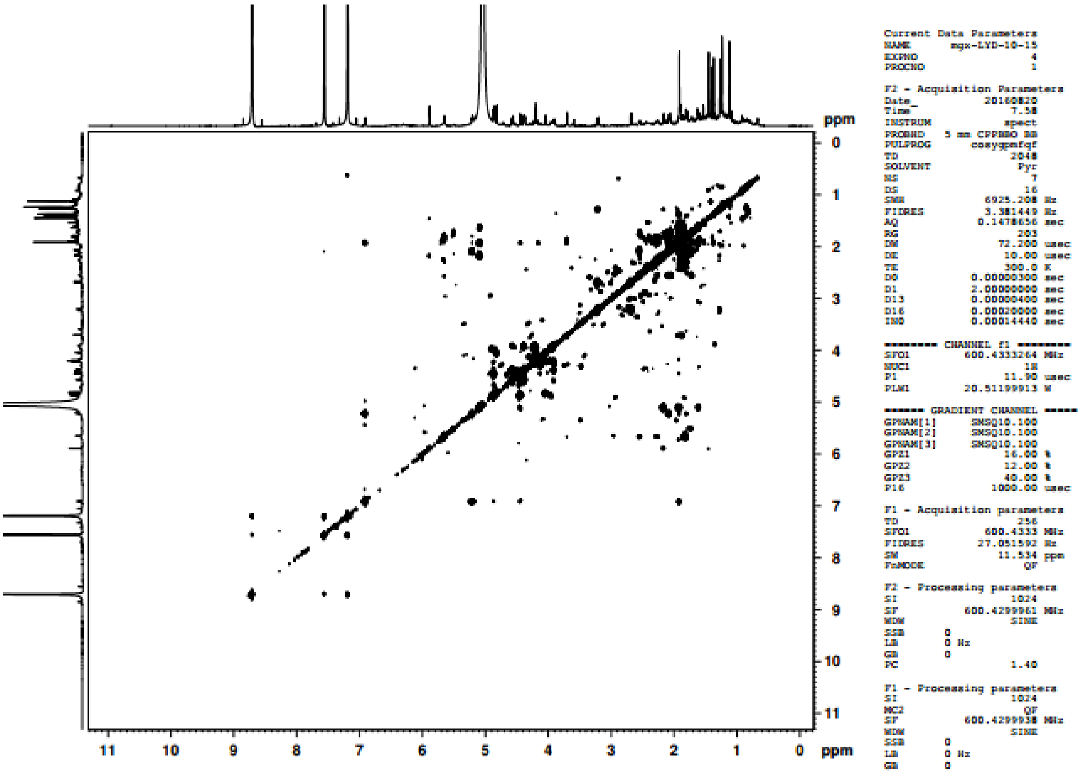

Figure S12. ^1H-^1H COSY spectrum of the new compound **2**

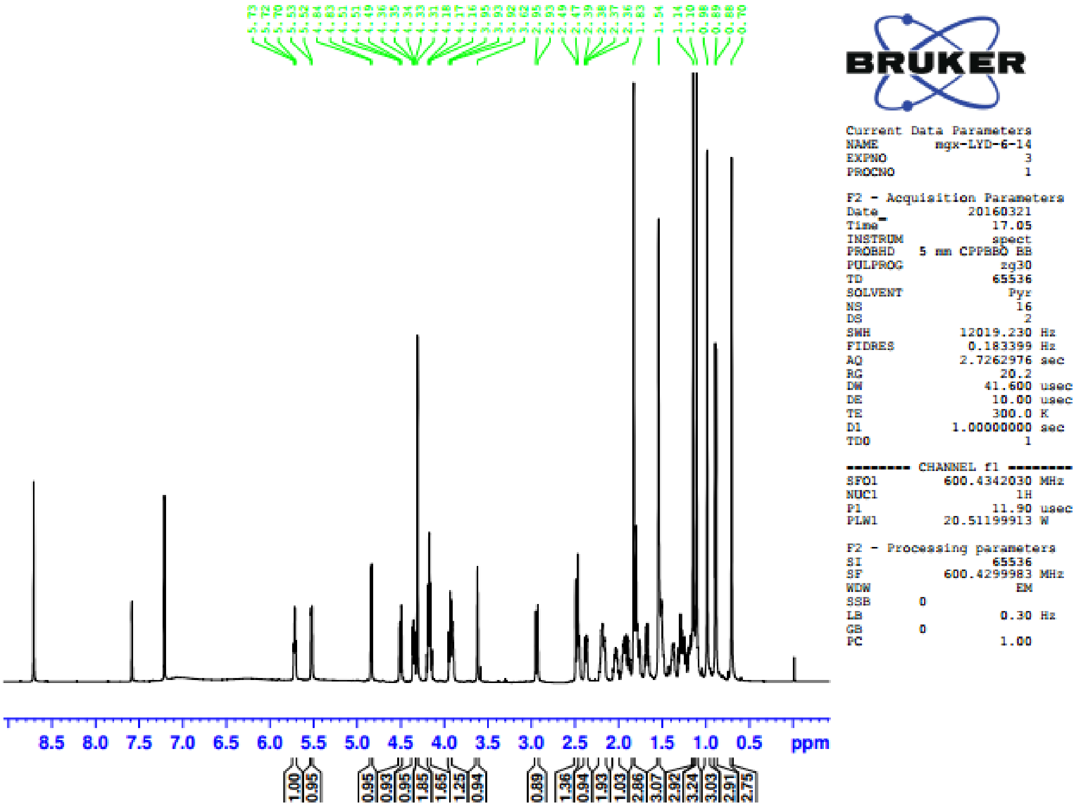

Figure S13. ^1H-NMR (600 MHz, pyridine-d_5) spectrum of the new compound **3**

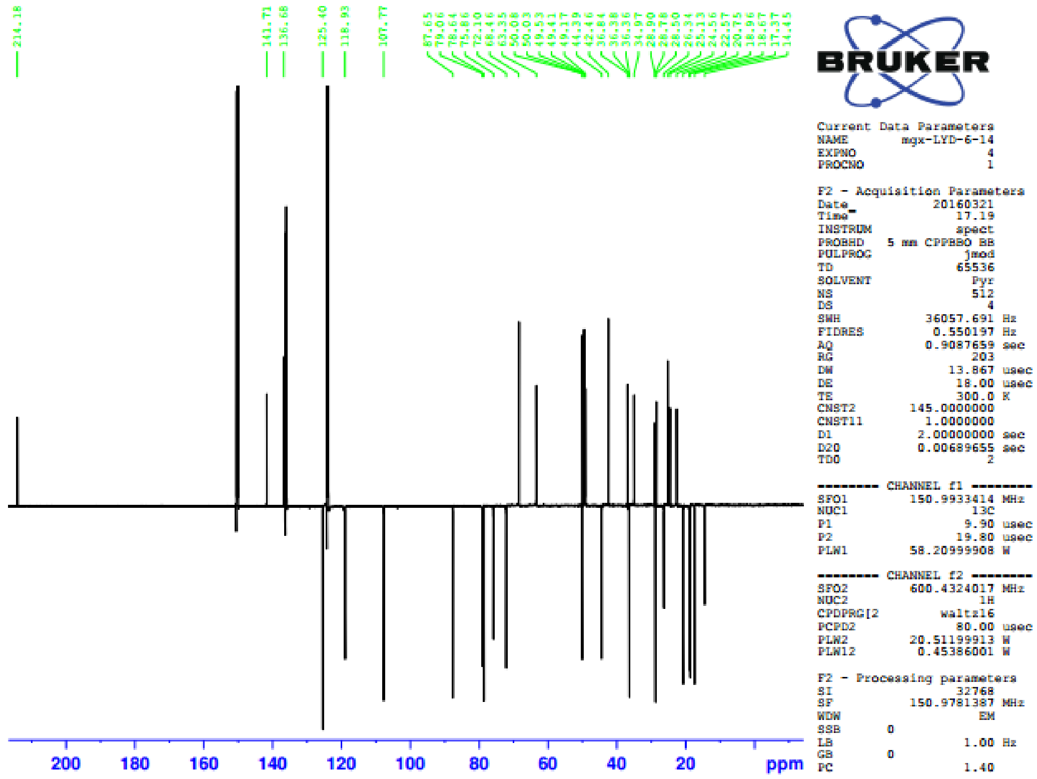

Figure S14. ^{13}C-APT (150 MHz, pyridine-d_5) spectrum of the new compound **3**

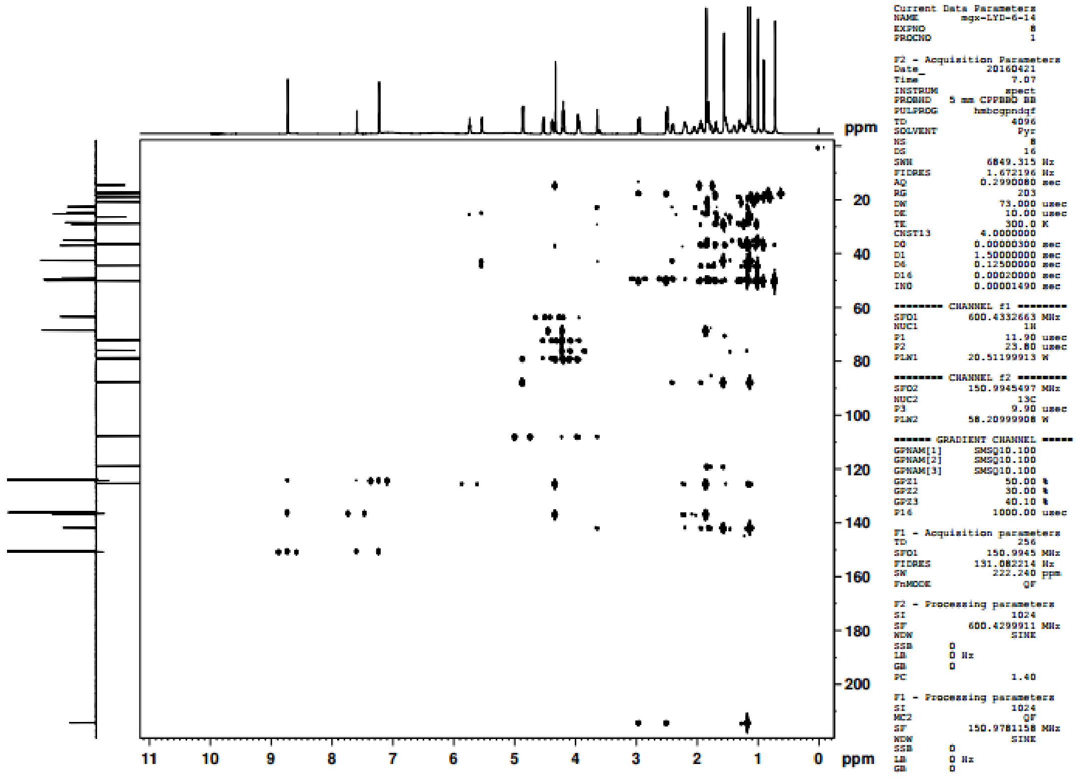

Figure S15. HMBC spectrum of the new compound **3**

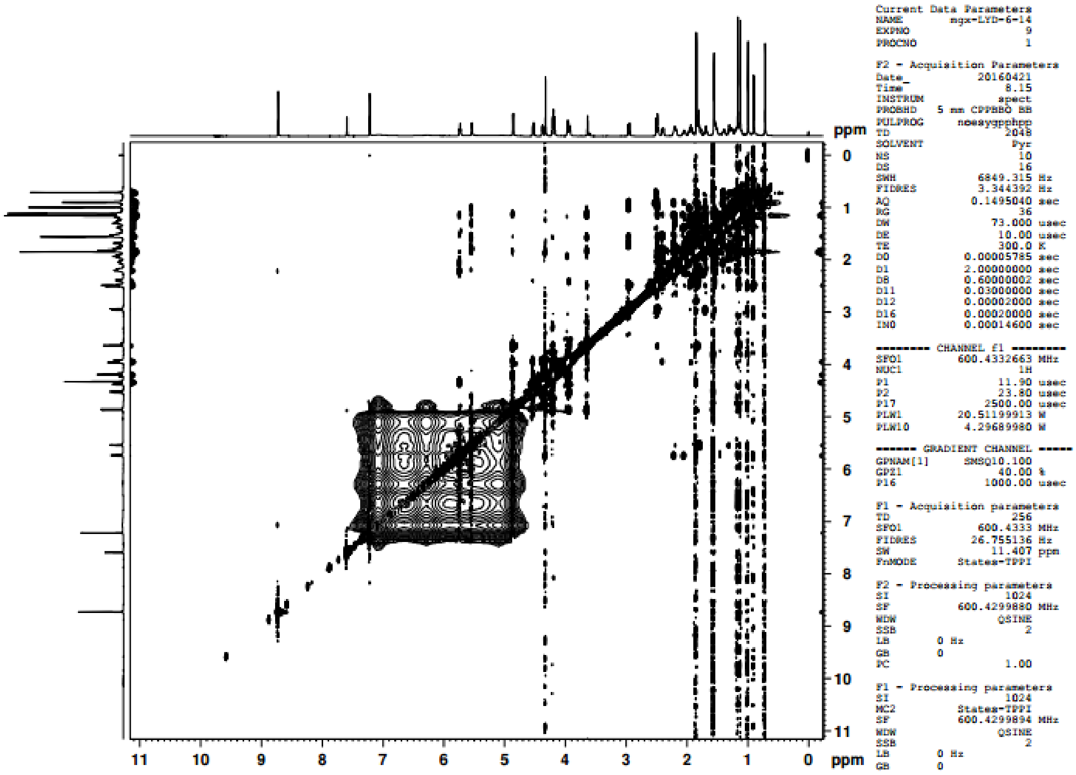

Figure S16. NOESY spectrum of the new compound **3**

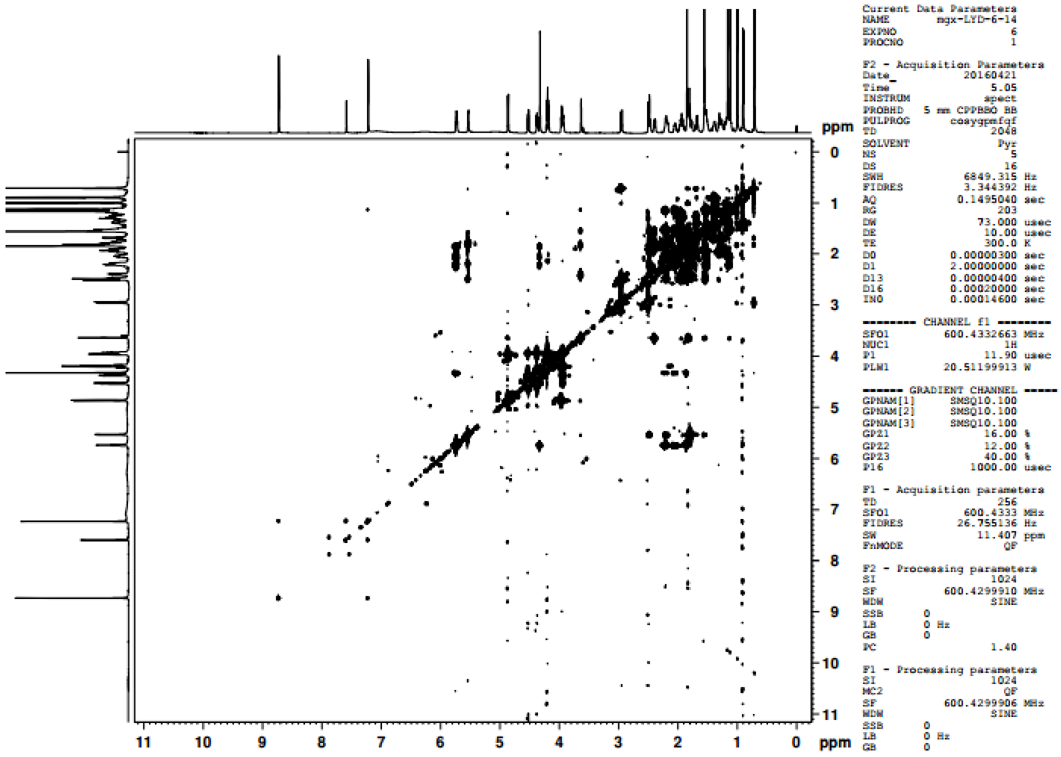

Figure S17. ^{1}H-^{1}H COSY spectrum of the new compound **3**

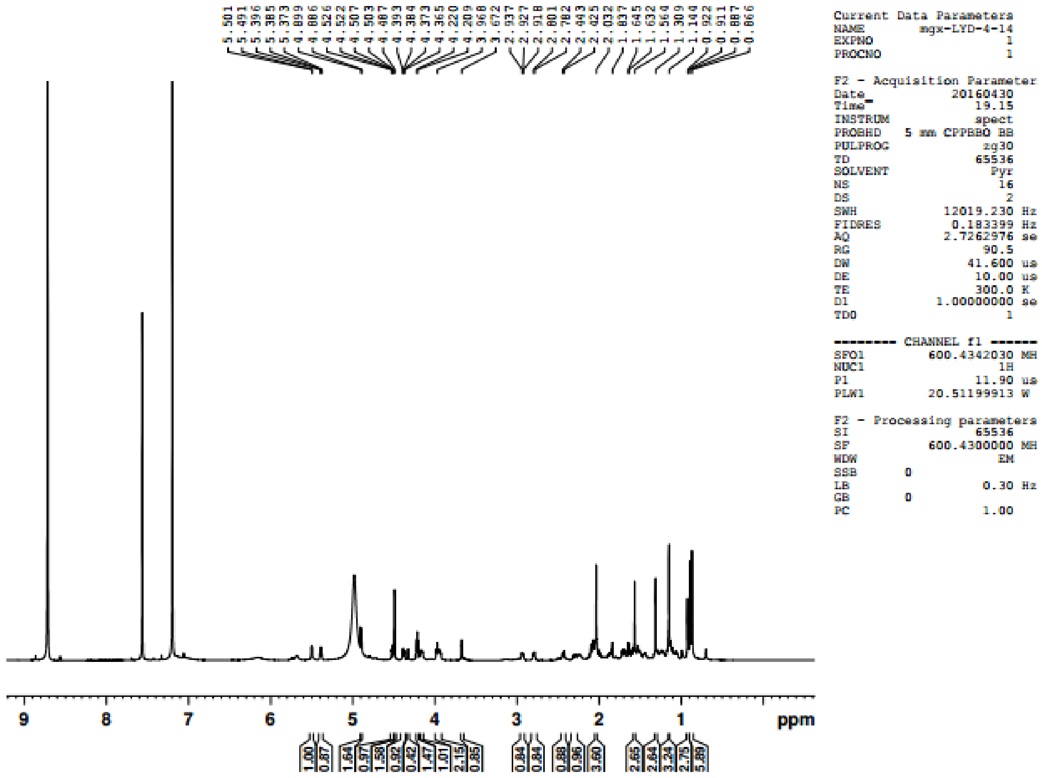

Figure S18. ^{1}H-NMR (600 MHz, pyridine-d_5) spectrum of the new compound **4**

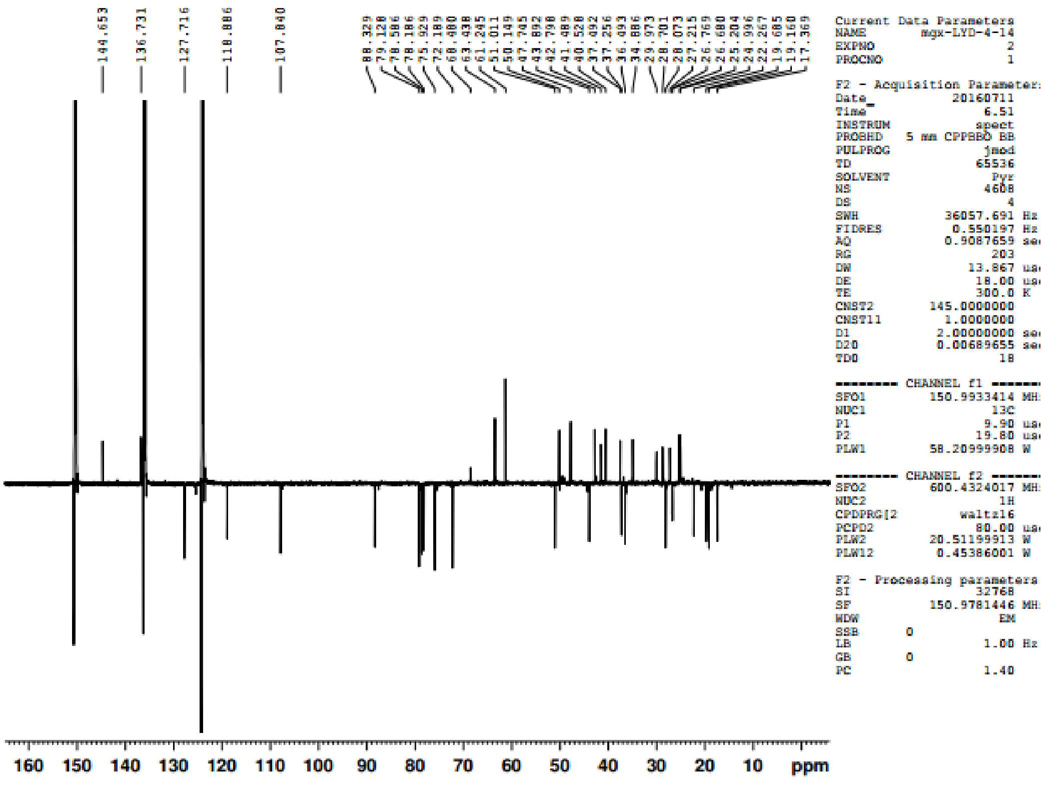

Figure S19. ^{13}C-APT (150 MHz, pyridine-d_5) spectrum of the new compound **4**

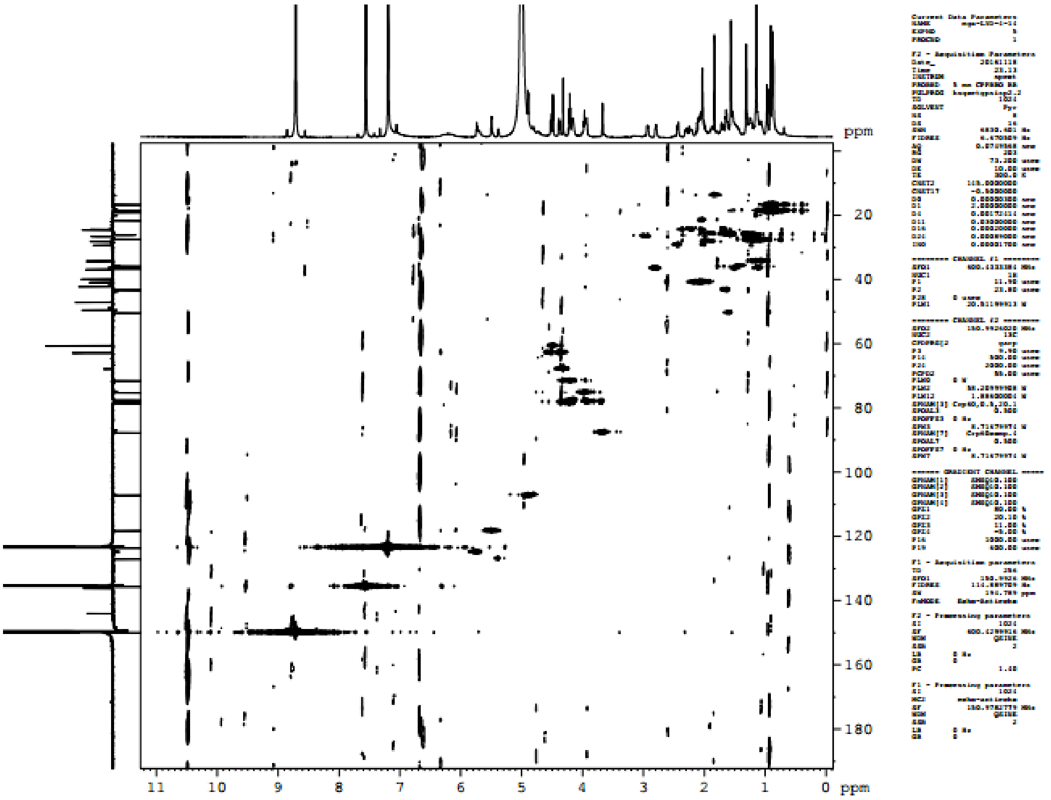

Figure S20. HSQC spectrum of the new compound **4**

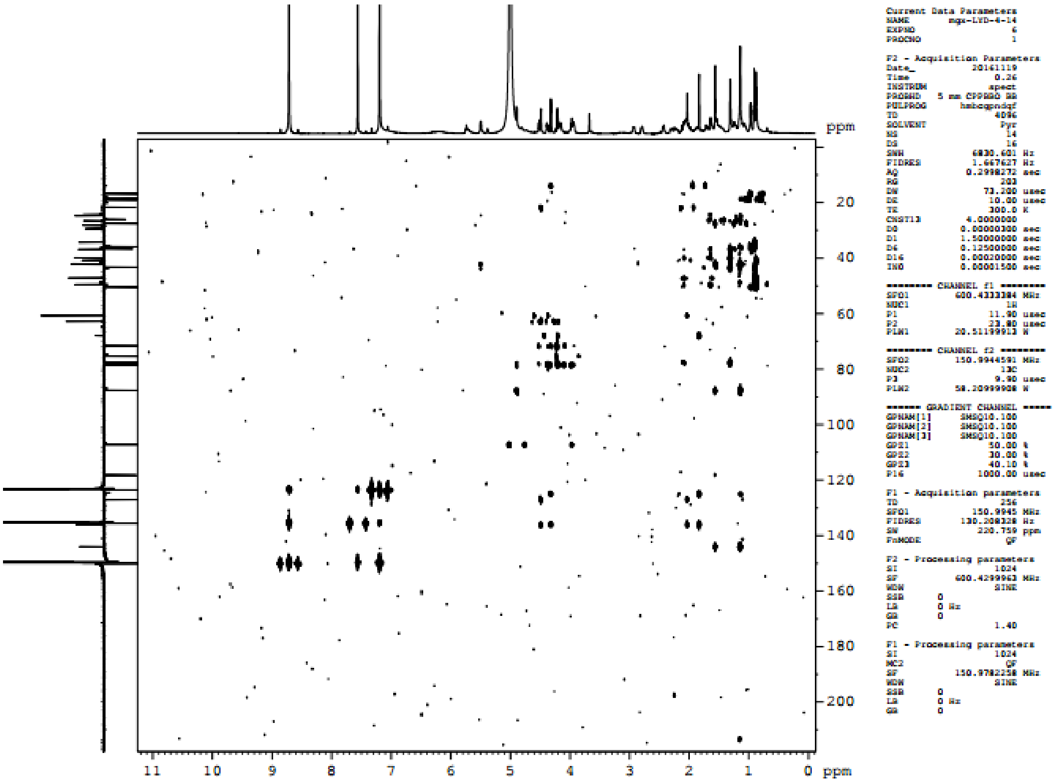

Figure S21. HMBC spectrum of the new compound **4**

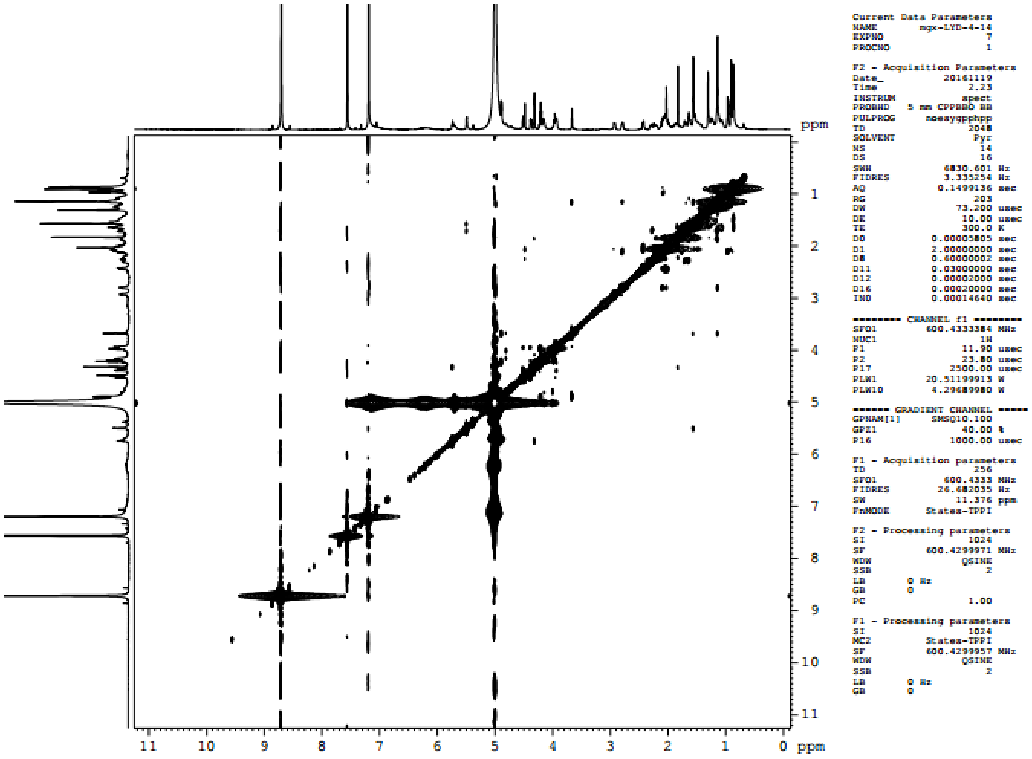

Figure S22. NOESY spectrum of the new compound **4**

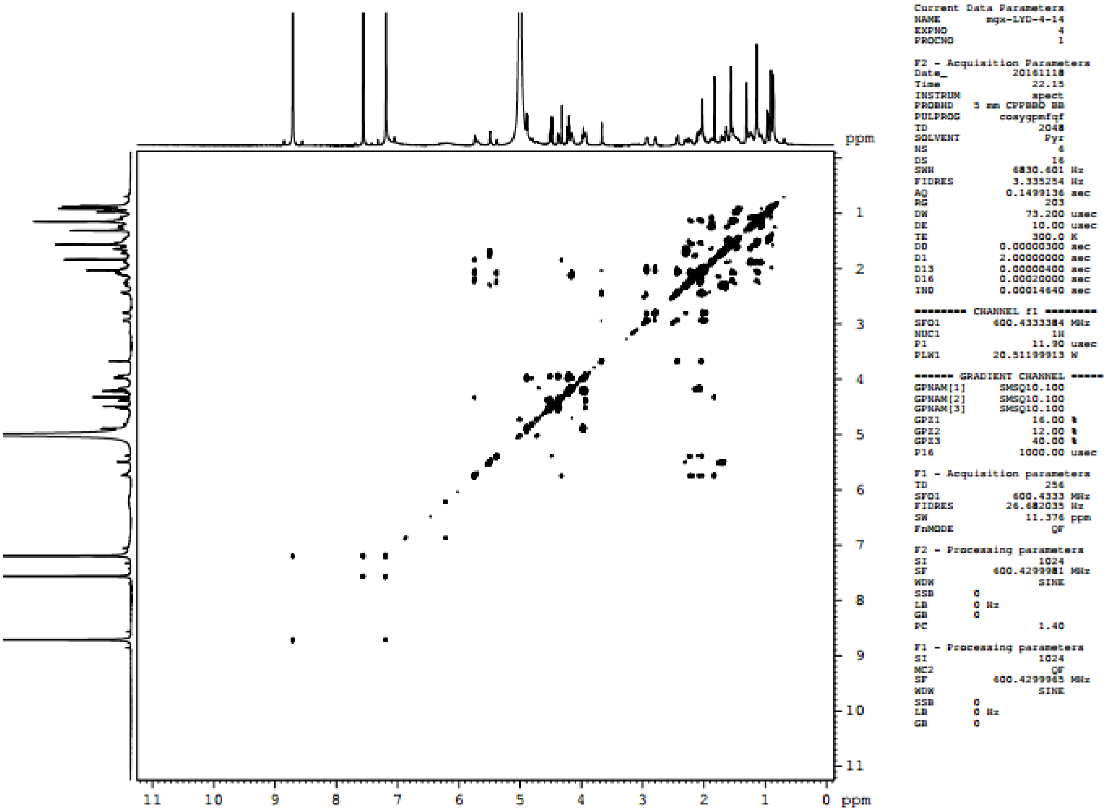

Figure S23. ^{1}H-^{1}H COSY spectrum of the new compound **4**

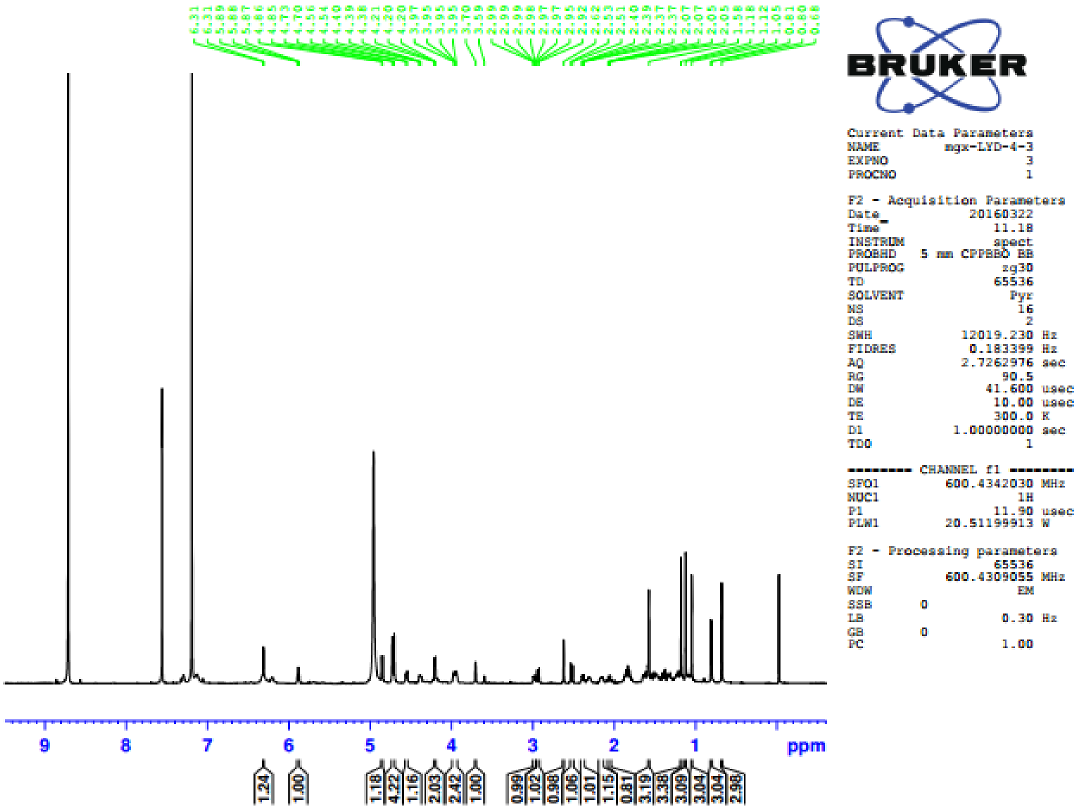

Figure S24. ^{1}H-NMR (600 MHz, pyridine-d_5) spectrum of the new compound **5**

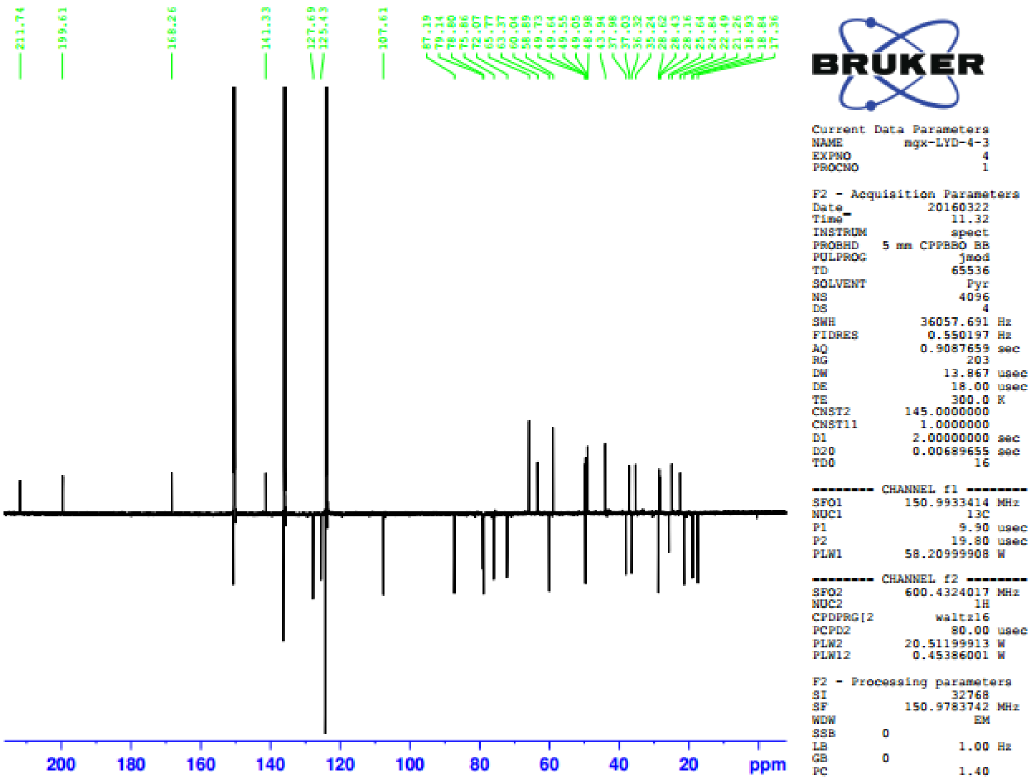

Figure S25. ^{13}C-APT (150 MHz, pyridine-*d*$_5$) spectrum of the new compound **5**

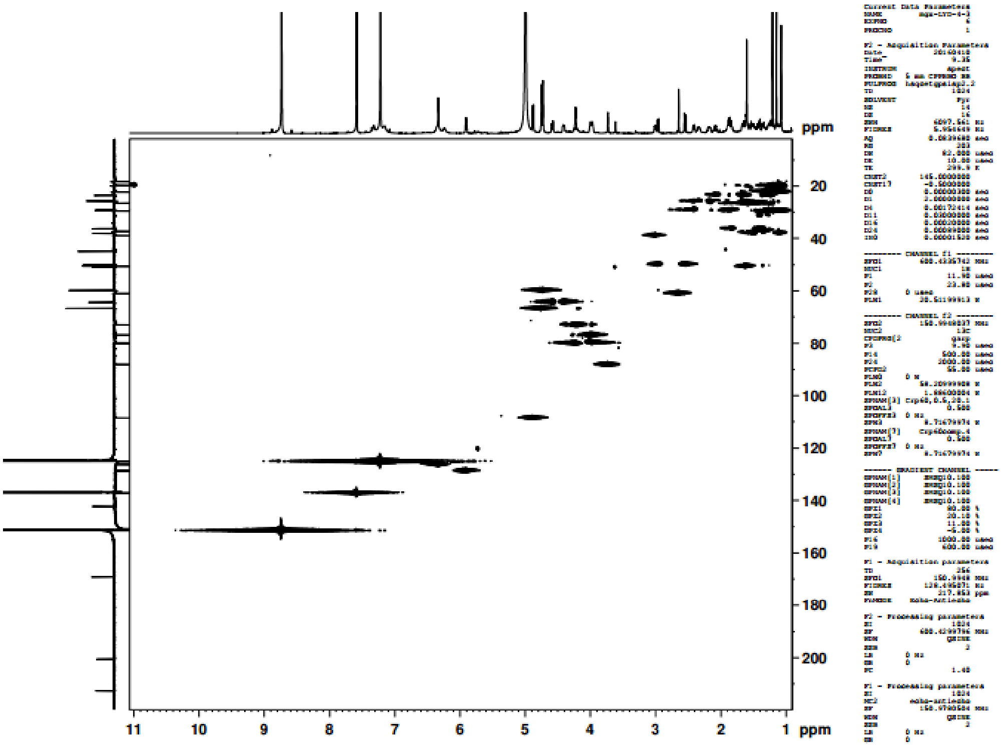

Figure S26. HSQC spectrum of the new compound **5**

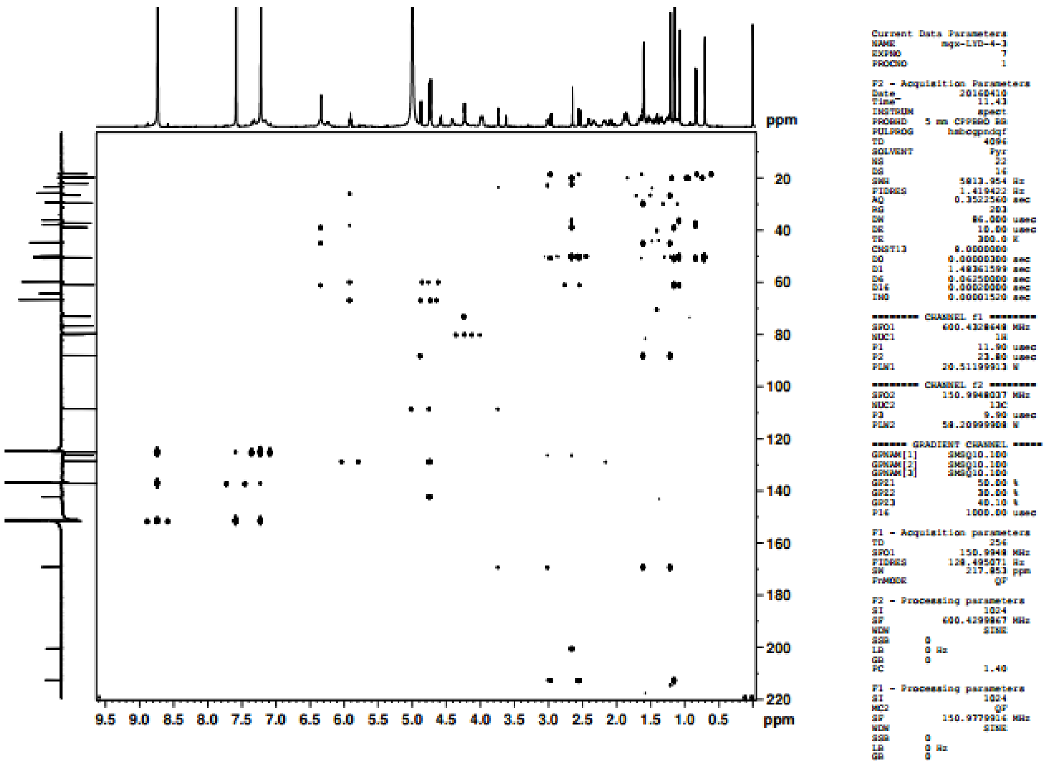

Figure S27. HMBC spectrum of the new compound **5**

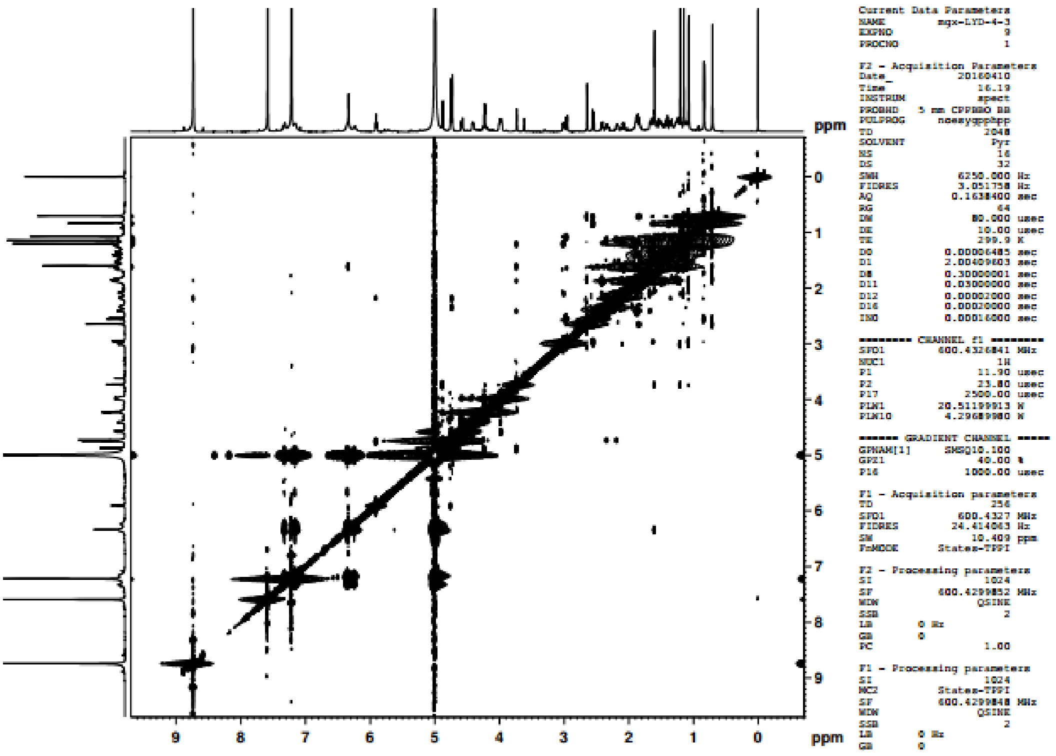

Figure S28. NOESY spectrum of the new compound **5**

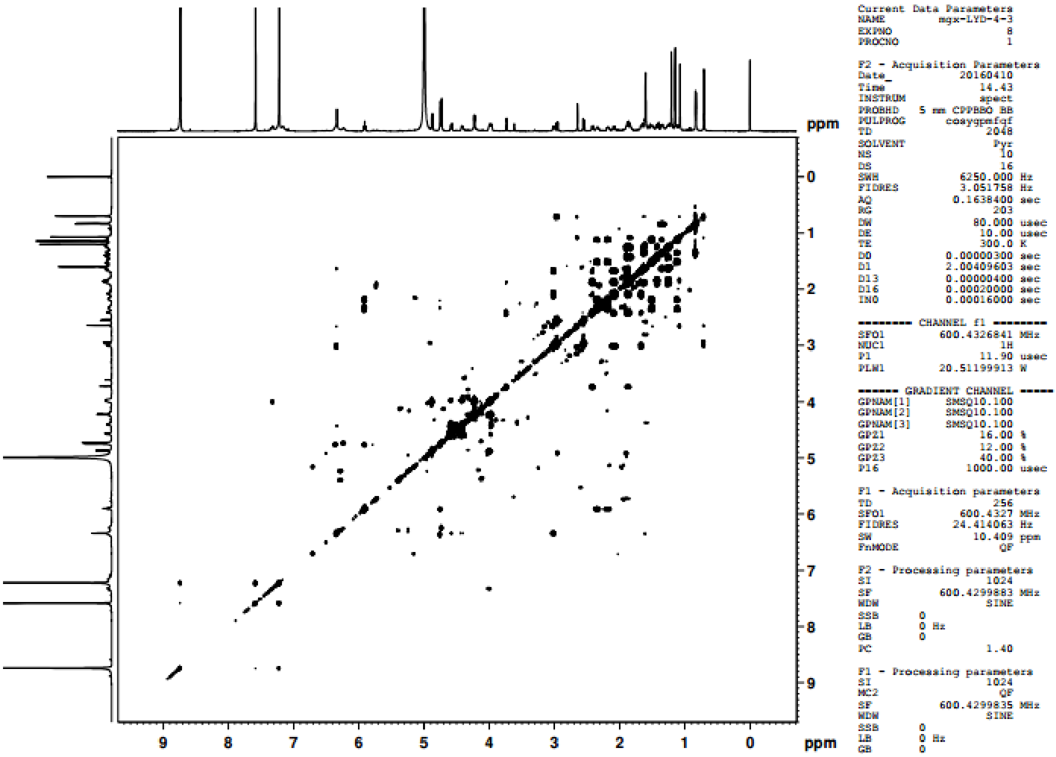

Figure S29. ^{1}H-^{1}H COSY spectrum of the new compound **5**
